# Water impacts nutrient dose responses genome-wide to affect crop production

**DOI:** 10.1038/s41467-019-09287-7

**Published:** 2019-03-26

**Authors:** Joseph Swift, Mark Adame, Daniel Tranchina, Amelia Henry, Gloria M. Coruzzi

**Affiliations:** 1 Center for Genomics and Systems Biology, Department of Biology, New York University, New York, 10003 NY USA; 2 Courant Institute for Mathematical Sciences, New York University, New York, 10012 NY USA; 3 International Rice Research Institute, Los Banos, 4031 Laguna, Philippines

## Abstract

Changes in nutrient dose have dramatic effects on gene expression and development. One outstanding question is whether organisms respond to changes in absolute nutrient amount (moles) vs. its concentration in water (molarity). This question is particularly relevant to plants, as soil drying can alter nutrient concentration, without changing its absolute amount. To compare the effects of amount vs. concentration, we expose rice to a factorial matrix varying the dose of nitrogen (*N*) and water (*W*) over a range of combinations, and quantify transcriptome and phenotype responses. Using linear models, we identify distinct dose responses to either *N*-moles, *W*-volume, *N*-molarity (*N*/*W*), or their synergistic interaction (*N*×*W*). Importantly, genes whose expression patterns are best explained by N-dose and W interactions (*N*/*W* or *N*×*W*) in seedlings are associated with crop outcomes in replicated field trials. Such N-by-W responsive genes may assist future efforts to develop crops resilient to increasingly arid, low nutrient soils.

## Abstract (web summary)

Scarcity of water and nutrients limit crop yields. Here Swift et al. show that gene expression in rice responds differently to changes in the absolute amount of nitrogen available compared to nitrogen concentration and identify expression profiles associated with crop performance in arid, low-nutrient soils.

## Introduction

Nitrogen (N) and water (W) are essential inputs for agriculture, however both are scarce in marginal soils, limiting crop production worldwide^1,2^. Since physiological studies show that N and W act synergistically to drive crop production^3–5^, we sought to discover how plants integrate responses to changes in N- and W-doses in rice—one of the world’s most important crops^6^.

While widely studied for their individual effects^7–12^, surprisingly little is known about how biological systems respond to combinations of N and W^13^. Since W serves as a solvent for N, we investigated whether plants respond to N as the absolute amount of N (*N*-moles) or the concentration of N in W (*N*-molarity). Plants are well-suited to address this basic question, because it is possible to vary N amount in a changing W environment (through soil drying), and results have practical relevance to crop production.

To this end, we present a factorial treatment matrix approach that systematically varies both N- and W-dose, and use linear equations to model how plants respond to *N*-amount, *W*-volume, and their interactions. Our linear models can distinguish between plant responses to changes in *N*-moles, *W*-volume, *N*-molarity (*N*/*W*), as well as a synergistic response *N*×*W*, both at the level of gene expression and phenotype. Moreover, gene sets responsive to *N*-moles, *W*-volume, or to the combined effect of N- and W-dose (*N*/*W* and *N*×*W*) are conserved across lab and field conditions. Importantly, the genes responding to combinations of N- and W-dose correlate with crop outcomes in the field over two seasons. Such genes that are specifically responsive to combinations of N- and W-dose can potentially be used as expression biomarkers to select rice varieties adapted to increasingly dry, nutrient-poor soils worldwide.

## Results

### Linear modeling uncovers the impact of W on N-dose responses

**Fig. 1:**
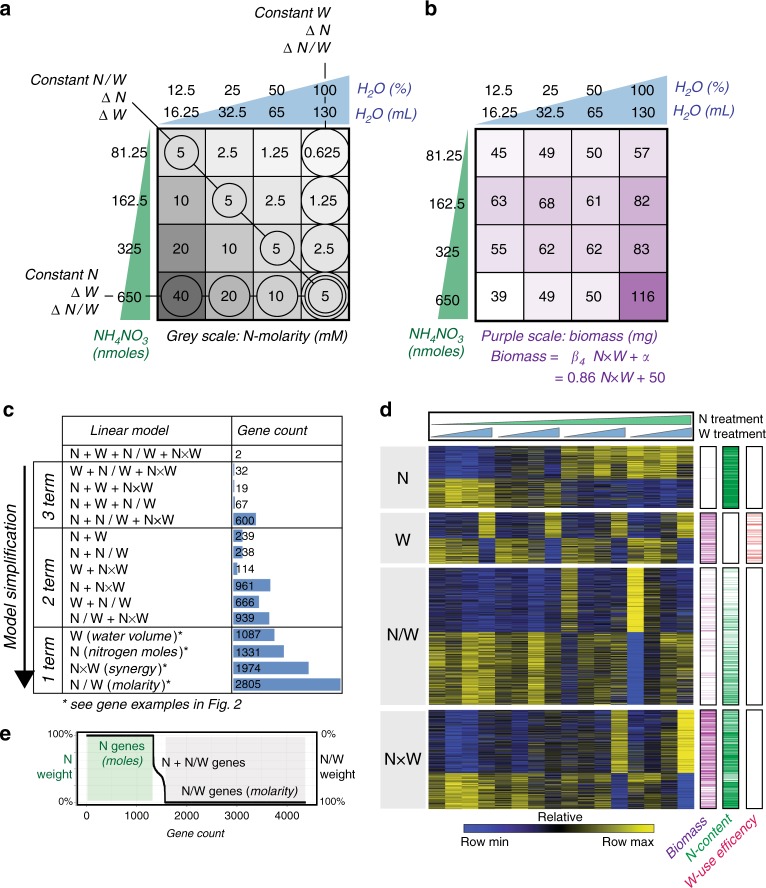
A factorial design uncovers plant responses to nitrogen and water dose combinations. **a** A 4-by-4 factorial matrix that varies both *N*-moles and *W*-volume can distinguish rice responses to *N*-moles, *W*-volume, and *N*-molarity. **b** A synergistic effect between *N*-moles and *W*-volume, modeled by the *N*×*W* interaction term, best explains changes in shoot biomass (linear model, *p* = 1.3 × 10^−5^). **c** Through model simplification, 14 linear models uncover genome-wide responses to *N*-moles, *W*-volume, *N*/*W* (molarity), *N*×*W*, and their combinations (linear model, adj. *p* < 0.005). **d** Expression heatmap of genes fitted by a single model term, and the proportion of genes within each class that significantly correlated with biomass, leaf N-content, and leaf W-use efficiency (Pearson correlation, adj. *p* < 0.05). **e** By comparing the normalized coefficients of genes fit by *N*, *N*/*W*, and *N* + *N*/*W* linear models, we found that genes were regulated exclusively in response to changes in *N*-moles (1331 genes) or *N*-molarity (2805 genes), while only 238 genes responded to a combination of changes in both *N*-moles and *N*-molarity. Source data for **d** are provided in Source Data file

To address how W impacts N-dose responses in plants, we designed a factorial N-by-W treatment matrix that could distinguish between plant responses to *N*-moles vs. *N*-molarity (Fig. 1a). Specifically, our 4-by-4 matrix of N-by-W treatments varied both the amount of *N*-moles (supplied as NH_4_NO_3_) and *W*-volume (modulated by soil drying). The highest amounts of N and W were chosen to promote plant growth, and the lowest amounts were chosen to limit plant growth. By varying *N*-moles and *W*-volume simultaneously, this matrix design allowed us to determine how rice plants respond to the total amount of N (*N*-moles), *W*-volume, or the concentration of N: *N*-molarity (*N*-moles/*W*-volume) (Fig. 1a, Supplementary Fig. 1).

To this end, 14-day-old rice seedlings initially grown on N- and W-replete media were transferred to each of the 16 treatment conditions within our N-by-W matrix (Fig. 1a). Plants were treated for 11 days, after which time we assayed leaf transcriptomes by RNA-seq and measured plant phenotypes (Fig. 1b, Supplementary Fig. 2). We note that our phenotypic measurements of plant *δ*^13^C, a proxy for W-use^14^, were proportional to the amount of external W provided (Supplementary Fig. 2). Similarly, we found total leaf *N*-content (a combination of assimilated ammonium and nitrate) was proportional to the amount of external *N*-moles provided (Supplementary Fig. 2). Together, these data indicated that internal leaf N- and W-status reflected their respective amounts in the external environment.

Phenotypically, we found that changes in shoot biomass in response to our N-by-W treatment combinations could be best modeled by a synergistic interaction of *N*-moles × *W*-volume (*N*×*W*) (Fig. 1b).

At the transcriptomic level, to interpret our gene expression data, we designed a multivariate linear model that could assess whether the expression of a given gene could be explained by a plant’s ability to respond to *N*-moles, *W*-volume, or their interaction, *N*-molarity (*N*/*W*). Based on the synergistic effects of *N*-moles and *W*-volume on biomass (Fig. 1b), we also included the *N*×*W* interaction term in our linear model (2), as detailed in Methods section. The benefit of using a linear model to interrogate our transcriptomic data was that it allowed us to detect gene expression responses that were directly proportional to changes in N- and/or W-dose.

We fit all expressed genes within the rice genome with this full linear model using DESeq2^15^, and through subsequent steps of model simplification, each gene could be binned into 1 of 14 simplified forms of the equation (Fig. 1c). We found that the expression of 65% of regulated genes (7197 genes) could best be explained by a single term—either *N*-moles, *W*-volume, *N*-molarity (*N*/*W*), or the synergistic interaction (*N*×*W*) (Fig. 1d, Supplementary Fig. 3). These four simple models of gene expression revealed that plants can respond to *N*-moles and *W*-volume independently from *N*-molarity (*N*/*W*). Moreover, they uncovered a synergistic response to *N*-moles and *W*-volume (*N*×*W*). The genome-wide evidence for how W impacts N-dose responses, and the role it plays in rice agriculture, is detailed below.

### Rice can respond independently to *N*-moles and *W*-volume

**Fig. 2:**
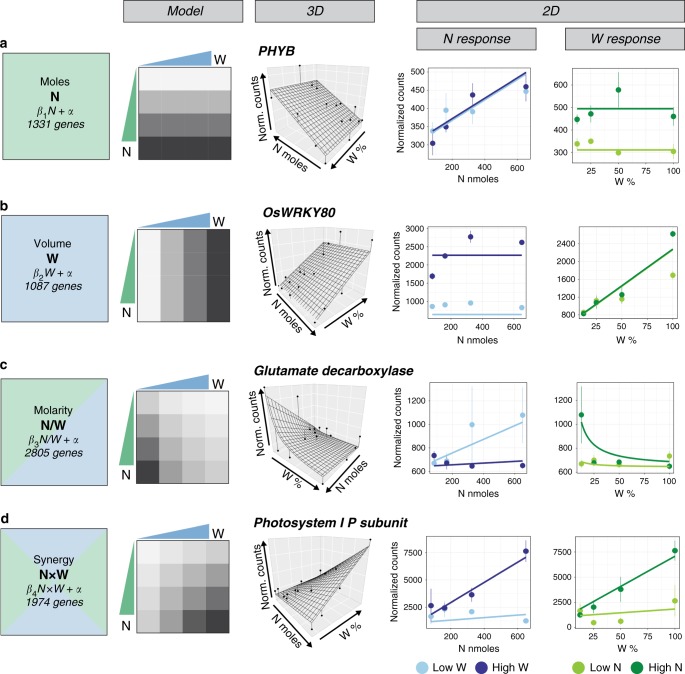
Linear modeling of genome-wide expression data uncovers four main responses to changes in *N*-moles and/or *W*-volume. **a** Expression of 1331 genes are dose-dependent on *N*-moles. *PHYB* expression, an example fitted by the *N*-moles model, is shown in three dimensions. *PHYB* is also plotted in two dimensions, showing its response to changes in *N*-moles under the lowest and highest *W*-volume provided (light blue: low W, dark blue: high W, error bars indicate SEM), and showing its response to changes in *W*-volume under the lowest and highest *N*-mole amounts provided (light green: low N, dark green: high N). **b** Expression of 1087 genes exhibit dose-dependent responses to changes in *W*-volume; *OsWRKY80* is an example. **c** Expression of 2805 genes exhibit dose-dependent responses to changes in *N*-molarity (*N*/*W*); *glutamate decarboxylase* is an example. **d** Expression of 1974 genes respond synergistically (*N*×*W*) to changes in N- and W-dose; *photosystem 1 P-subunit* is an example. Figure 2 source data are provided in Source Data file

Our modeling of genome-wide responses revealed that rice plants can respond independently to the amounts of *N*-moles or *W*-volume available within the treatment media. We found that 1331 rice genes responded exclusively to changes in *N*-moles in a dose-dependent manner, independently of *W*-volume (Fig. 2a, Supplementary Data 1). This class of genes displayed changes in expression that were best explained by changes in *N*-moles, and contained known N-responsive genes involved in N-uptake and assimilation such as the ammonium transporter *OsAMT1*^11^ and *glutamate synthase* (*GOGAT*)^16^. It also contained genes not previously known to be N-regulated, such as phytochrome *PHYB*, a light sensor and signal transducer^17^ (Fig. 2a). This *N*-mole responsive gene class was over-represented in N-relevant gene ontology (GO) terms such as N-compound transport, and N-assimilation (Supplementary Data 2). Additionally, the vast majority of genes within this class (93%) significantly correlated with changes in leaf N-content (Fig. 1d).

Separately, we identified 1087 rice genes that specifically responded to changes in *W*-volume in a dose-dependent manner, independently of *N*-moles (Fig. 2b, Supplementary Data 1). This set included genes implicated in drought responses, including rice orthologs of *Arabidopsis* genes involved in abscisic acid signaling (*ABF2*)^18^ and biosynthesis (*AAOs 1-4*)^19^, as well as *OsWRKY80*, a member of the WRKY transcription factor family involved in W responses^20^ (Fig. 2b). Furthermore, genes within this *W*-volume response class significantly correlated with changes in leaf W-use efficiency measurements (*δ*^13^C) (Fig. 1d).

### Rice responds to interactions between *N*-moles and *W*-volume

Our genome-wide models of transcriptome data also uncovered sets of genes whose dose-dependent response to *N*-moles was impacted by *W*-volume. The expression of these genes could be modeled by an interaction between *N*-moles and *W*-volume—*N*-molarity (*N*/*W*) or synergistically (*N*×*W*)—as described below.

Our analysis uncovered 2805 genes whose expression patterns were best explained by a change in *N*-molarity (*N*/*W*) (Fig. 2c, Supplementary Data 1). This set of genes was significantly enriched in N-related GO terms including cellular N-compound biosynthetic processes (Supplementary Data 2). Among these genes were the N-assimilation genes *aspartate aminotransferase*^21^ and *glutamate decarboxylase* (Fig. 2c).

Thus, our models of genome-wide responses to N and W revealed that plants can respond to dose changes in *N*-moles (Fig. 2a), *W*-volume (Fig. 2b), or *N*-molarity (Fig. 2c). Moreover, we found that genome-wide responses to changes in *N*-moles (1331 genes) vs. *N*-molarity (2805 genes) were largely dichotomous; only 238 genes were sensitive to both (Fig. 1e).

Importantly, we found that W could also impact genome-wide N-dose responses in a synergistic mode. Specifically, we uncovered 1974 genes whose expression patterns could best be explained by a synergistic response to *N*-moles and *W*-volume (*N*-moles × *W*-volume) (Fig. 2d, Supplementary Data 1). Fifty-four percent of these genes significantly correlated with changes in biomass of rice seedlings—the highest proportion of any gene class (Fig. 1d). Upregulated genes within this *N*×*W* synergistic class were enriched in GO terms related to growth and energy production, such as photosynthesis and ATP biosynthetic processes, and members included seven ribosomal subunits and the rice ortholog of the *Arabidopsis photosystem I P-subunit* (Fig. 2d, Supplementary Data 2).

Thus, synergistic gene responses to changes in *N*-moles and *W*-volume may be a means by which plants signal growth responses when absolute amounts of both N and W are optimal. Such gene expression responses that are dependent on a multiplicative, nonlinear interaction between N and W can ensure that linear changes in both N and W amounts have non-additive outcomes on gene expression levels and plant phenotypes.

### N-responsive gene classes respond rapidly to N-dose

**Fig. 3:**
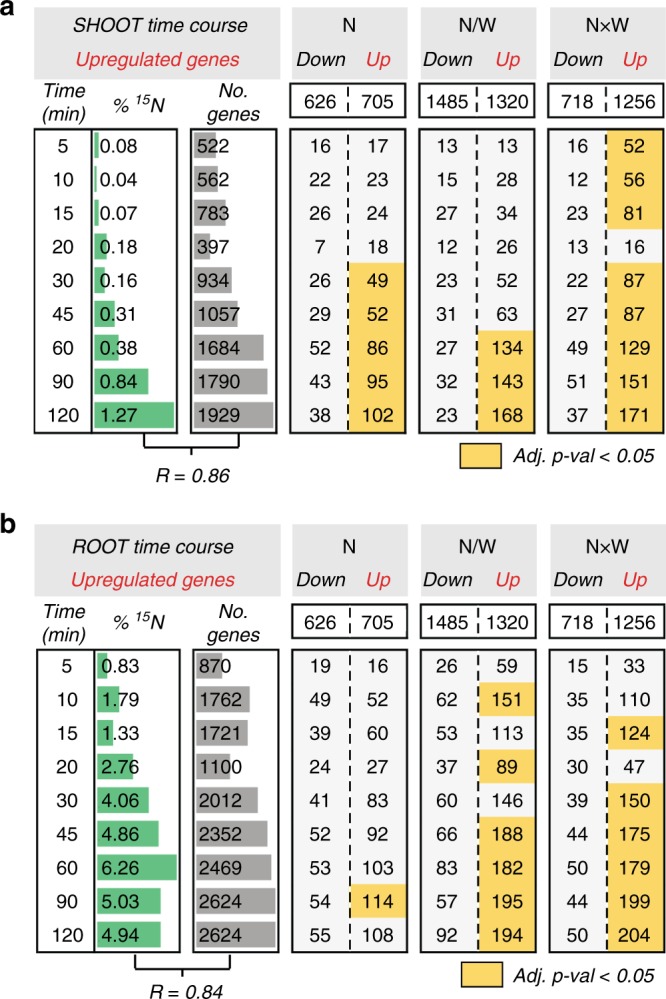
All three N-responsive gene classes rapidly respond to a change in N-dose. **a** Rice seedlings were treated with a N-dose for 120 min (see Methods). During this period, we monitored shoot gene expression responses and shoot N-uptake (via ^15^N labeling). ^15^N-uptake significantly correlated with the total number of differentially expressed N-response genes (Pearson *R* = 0.86, *p* = 2.8 × 10^−3^). The table shows that the number of genes found in *N*, *N*/*W*, and *N*×*W* classes that were upregulated by N within our factorial N-by-W matrix experiment intersect significantly with the number of genes upregulated in response to a change in N-dose in the time-course experiment (Monte Carlo, adj. *p* *<* 0.05). **b** The same analysis as in **a** was performed in root tissue (Pearson *R* = 0.84, *p* = 4.3×10^−3^). Overlaps of downregulated N-response genes are presented in Supplementary Fig. 4. Figure 3 source data are provided in Source Data file

To address whether responses to *N*-moles, *N*/*W*, and *N*×*W*, occur due to changes in nutrient dose, rather than being the product of plant stress or long-term changes in plant physiology, we investigated how rapidly these gene sets respond to changes in N-dose under non-stress, W-replete conditions. To do this, we performed a time-course experiment, treating hydroponically grown rice seedlings for 2 h with an N-treatment (provided as 20 mM K^15^NO_3_ + 20 mM ^15^NH_4_^15^NO_3_). Over 10 time points, we monitored transcriptomic responses, as well as ^15^N-uptake into root and shoot tissue. We detected differentially expressed genes using a cubic spline model, and then binned genes into each time point they were found upregulated or downregulated, defined as when their fold change passed a threshold of ±1.25. In both root and shoot tissues, the number of differentially expressed genes found in each time point significantly correlated with the level of ^15^N incorporation, supporting the notion that such N-dose responsive genes were the result of N-influx from the environment (Fig. 3a, b, Supplementary Fig. 4, Supplementary Data 3).

Our time-course study revealed that all three classes of N-dose responsive genes—*N*-moles, *N*/*W*, and *N*×*W*—respond rapidly (within 5–30 min) to a change in N-dose under W-replete conditions. This was revealed by overlapping genes found to be regulated within each time point of the N-treatment with each of the three N-dose responsive gene classes defined from the N-by-W matrix (Fig. 3a, b, Supplementary Fig. 4). In both root and shoot tissue, we found that genes within each N-dose response class found in the N-by-W matrix experiment—*N*-moles, *N*/*W*, and *N*×*W*—were significantly enriched within the N-treatment time-course experiment. Furthermore, the direction of gene regulation in response to N-dose (either repression or activation) was conserved between experiments (Fig. 3a, b, Supplementary Fig. 4). Collectively, these data indicate that N-dose response genes within each class—*N*-moles, *N*-molarity (*N*/*W*), and synergistic (*N*×*W*)—respond rapidly to changes in N-dose under non-W-stress conditions.

### Nonlinear N-by-W gene responses correlate with field traits

As the distinct modes by which W informs N-dose responses were discovered in rice seedlings, we next asked whether these gene responses play a role in rice agriculture.

**Fig. 4:**
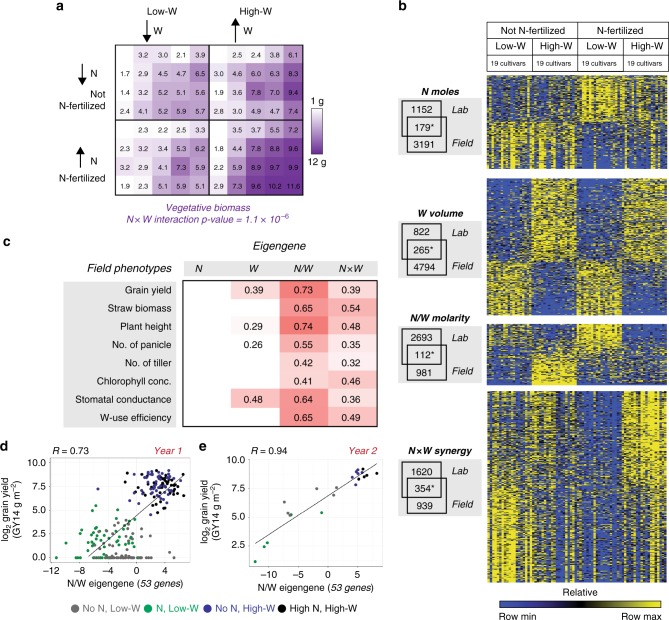
Genes responding nonlinearly to combinations of N-fertilizer and W treatment are associated with agricultural outcomes. **a** Nineteen rice cultivars were grown in the field under a 2-by-2 matrix varying N-fertilizer and W treatment; each cell indicates the average biomass of each of the 19 cultivars. The synergistic interaction *N*×*W* could best explain differences in shoot biomass (three-way analysis of variance interaction term, *p* = 1.1 × 10^−6^, *F* = 24.6, df = 1). **b** The genes responding to combinations of N- and/or W-dose in rice seedlings found under laboratory conditions overlap significantly with reciprocal classes found in field-grown plants (*Monte Carlo, *p* < 0.05). Normalized expression patterns of lab field-validated genes are displayed in heatmap. **c** Eigengenes derived from each gene set were correlated with crop traits. Significant Pearson *R* values are shown in red (permutation test, *p* < 0.05). **d** Example from **c**. Changes in *N*/*W* eigengene expression across 228 field samples is associated with grain yield (gray: −N, −W, green: +N, −W, blue: −N, +W, black: +N, +W conditions). **e** *N*/*W* eigengene expression is predictive within an independent field test set. Source data for **b**–**e** are provided in Source Data file

To answer this, we grew 19 different rice cultivars in the field at the International Rice Research Institute (IRRI) in the Philippines. Each rice cultivar was grown in a 2-by-2 factorial matrix that varied N-fertilizer dose and *W*-volume. Crops were N-fertilized at a high dose of 150 kg/ha, or not fertilized. Under each N-dose condition, crops were grown either under W-replete vs. -deplete conditions, creating well-irrigated or drought conditions (Fig. 4a, Supplementary Fig. 5). For the 228 samples generated, we assessed vegetative and yield phenotypes, and took vegetative leaf samples for RNA-seq analysis.

In agreement with our laboratory observations on rice seedlings (Fig. 1b), we found that a synergistic interaction between N-fertilizer amount and W treatment (*N*×*W*) could best explain rice vegetative biomass in mature plants grown under field conditions (Fig. 4a).

To investigate whether the same N-by-W gene expression patterns we identified under laboratory conditions (Figs. 1 and 2) were present under field conditions, we assessed whether genome-wide expression patterns in rice field samples could be explained by N-fertilizer amount, W treatment, or the interaction between the two (where genotype was treated as a covariate within the model). By these means, we found that the genome-wide expression patterns across 19 rice cultivars tested in the field were consistent with the four classes of nutrient dose responses we discovered in seedlings under laboratory conditions—*N*-moles, *W*-volume, *N*-molarity (*N*/*W*), or synergistic responses (*N*×*W*) (Fig. 4b, Supplementary Data 4). Moreover, we found that the overlaps of lab-field gene sets for each nutrient dose responsive gene class were significantly higher than expected by chance (Fig. 4b).

Next, we investigated whether expression levels of dose responsive genes that were directionally conserved (i.e. induced or repressed) in each gene class across both laboratory and field settings were associated with cultivar agronomic performance. To do this, for each gene class set—*N*, *W*, *N*/*W*, and *N*×*W* —we calculated the first principal component, which represented the expression trends of all gene members in a single profile or ‘eigengene’ ^22^. Each resulting eigengene thus represented the set of lab-field validated genes responding either to *N*-moles (56 genes), *W*-volume (176 genes), *N*-molarity (*N*/*W*) (53 genes), or the synergistic interaction between N and W dose (*N*×*W*) (174 genes) —where each eigengene accounted for 31, 23, 32, and 34% of the proportion of variance in gene expression, respectively. We then assessed whether the expression profile for each of the eigengenes representing the four gene classes correlated with field phenotypes. The significance of this association was calculated by comparison to a null distribution of 10,000 eigengenes, generated from randomly selected genes expressed in the field.

This eigengene analysis revealed that the expression of genes regulated nonlinearly in response to changes in combinations of N- and W-doses—*N*/*W* and *N*×*W*—were significantly associated with traits important to crop production (Fig. 4c, Supplementary Fig. 6). Genes responding synergistically to changes in N- and W-doses (*N*×*W*) or to *N*-molarity (*N*/*W*) across the lab-field divide were significantly correlated with complex traits such as grain yield, straw biomass, plant height, number of panicles, and number of tillers (Fig. 4c). Moreover, they were associated with N- and W-dose related traits such as chlorophyll concentration, stomatal conductance, and W-use efficiency (Fig. 4c).

Our findings that *N*/*W* and *N*×*W* gene sets, identified in both lab-grown seedlings and field-grown mature plants, were associated with final grain yield could be of particular use to rice breeders. To confirm that expression of these N-dose response gene sets are associated with yield, we repeated our field experiment the following year (Supplementary Fig. 7), and sequenced the transcriptomes of two genotypes that varied in their yield outcomes. Through repeating our eigengene analysis on this independent field data test set, we validated that genes whose expression patterns is best explained by changes in N and W combinations—*N*/*W* or *N*×*W*—were significantly associated with grain yield (Fig. 4d, e, Supplementary Fig. 8).

## Discussion

Our linear modeling of genome-wide expression patterns provide insight into how W impacts nutrient dose responses in a biological system. Typically, nutrient response studies vary nutrient dose within a fixed volume of W. However, this design changes both nutrient amount and concentration, making it impossible to determine whether nutrient responses are governed by the absolute nutrient amount, or its concentration in W. By systematically varying both N- and W-dose, we were able to discriminate between plant responses to N amount (moles) and N-concentration (molarity). Furthermore, we found that genes that respond nonlinearly to N- and W-dose combinations have relevance to crop production.

The design of our factorial N-by-W matrix enabled us to distinguish, measure, and model plant responses to changes in N-dose as a function of W (Fig. 1a). Our linear models of transcriptome and phenotype data could explain plant responses to changes in N-dose according to one of three distinct modes. First, plants can respond to changes in *N*-moles, independently of *W*-volume. In addition, plants can respond to changes in N-dose as a function of *W*-volume in one of two ways, as *N*-molarity (*N*/*W*), or synergistically (*N*×*W*).

Importantly, the ability of plants to respond to N-dose as a function of W has practical relevance to crop production. Indeed, the genome-wide responses to N-by-W interactions (*N*/*W* or *N*×*W*) that we observed in rice seedlings grown under controlled laboratory conditions correlated with agricultural traits in mature plants measured in the field over two seasons. Specifically, genes regulated in response to changes in both N and W (*N*/*W* or *N*×*W*) correlated with field traits such as yield, straw biomass, height, panicle number, tiller number, chlorophyll concentration, stomatal conductance, and W-use efficiency (Fig. 4c). As such, genes that exhibit N-by-W dose responses are associated with the non-additive effects irrigation and N-fertilizer have on crop phenotype.

A list of the genes responding to N-by-W dose interactions (*N*/*W* or *N*×*W*) that are associated with crop outcomes are reported in Supplementary Data 5. These N-by-W responsive gene sets could be useful as expression biomarkers to select rice varieties adaptive to marginal soils that are both N and W poor^1,23^. Because these genome-wide responses detected at the seedling stage were associated with crop outcomes in mature field-grown rice, they may potentially be used to accelerate molecular breeding studies. Additionally, these N-by-W responsive genes could potentially serve as a biological readout for how N availability changes with W status within the rhizosphere, since measuring the effects of W on N flux within soils through existing chemical assays is technically challenging.

We note that these N-by-W effects that we measured on above ground crop traits are highly dependent on roots, which govern both nutrient and W uptake from soil^24^. Indeed, decreasing W content in soil not only increases *N*-molarity but also leads to changes in root physiology, which alters the plant’s ability to acquire nutrients. Root physiological responses to W deficit that may negatively affect nutrient uptake include development of apoplastic barriers that prevent root W loss^24,25^, and the development of root aerenchyma^26,27^. However, some root physiological responses to W deficit may also improve nutrient uptake, such as increased root foraging^28^. Thus, future studies should investigate how root physiology informs the combinatorial responses to N and W we report here. However, at the gene expression level, we note that plants with very different root systems (i.e. seedlings vs. mature field-grown plants) share the same N-by-W dose response genes in shoots (Fig. 4b). Additionally, our data suggest that some of the N-by-W response genes detected in shoots also occur in roots (Fig. 3b, Supplementary Fig. 4).

How such changes in N- and/or W-dose can be sensed or integrated at the molecular level (as *N*/*W* or *N*×*W*) is unknown. N-dose sensing may involve proteins involved in N-uptake, such as transceptors of nitrate or ammonia^29,30^, or sensors of internal N-status^8^. We note that the exact sensed N species and N-sensor(s) remain under investigation across eukaryotes^31,32^. The ability to sense changes in N-by-W doses may rely on systemic signaling interactions—the product of integrated biological processes occurring at the organismal level^33^. Such integration at the whole plant level may be achieved through cross-talk between N-signaling and the W-responsive hormone ABA^9^, mediated via transporters^34^, or transcriptional regulators responsive to both signals^35^.

Finally, since the combinatorial effects between nutrients and W are seen in crop phenotypes across agriculture^3–5^, future work should also investigate whether the gene regulatory responses found here hold for other crop species and other types of nutrients. Similarly, since the genes governing nutrient-sensing responses are conserved across biology^31^, the impacts of W on nutrient dose responses that we have uncovered in plants should now be investigated in other biological systems.

## Methods

### Plant growth treatments and phenotypic assays

We grew rice seedlings within a 4 × 4 factorial treatment matrix varying nitrogen (N) and water (W) doses (Fig. 1a). To create these treatment conditions, rice seedlings (*Nipponbare*) were first grown for 2 weeks on Yoshida media, supplemented with 5 mM NH_4_NO_3_^36^, under 12 h light (150/µmol^2^/s)/12 h dark diurnal cycle, at temperatures 27 and 25 °C respectively and 70% humidity. Endosperms were then removed, and three plants were transferred to each pot containing 680 g of sand, which could hold a maximum of 130 mL of W.

To create distinct N-dose conditions, each pot contained 130 mL of one of four NH_4_NO_3_ concentrations (0.625, 1.25, 2.5, and 5 mM) in Yoshida media. One percent of N atoms were labeled either as ^15^NH_4_ or ^15^NO_3_. Plants were maintained at complete pot saturation (130 mL) for 3 days. To create distinct W-doses, W was allowed to evaporate off pots (Supplementary Fig. 1). Once the target W-saturation was reached, saturation was maintained by daily additions of W. In this way, four *W*-volumes were achieved—16.25, 32.5, 65, and 130 mL—which corresponded to four W-saturation levels—12.5, 25, 50, 100%. Each N by W dose treatment combination was tested in triplicate, resulting in 48 samples for RNA-seq analysis.

After 11 days of N and W treatment, a portion of the youngest mature leaf from three rice seedlings from each treatment condition was sampled for transcriptomic analysis 2 h after subjective dawn. At this time, we also recorded plant shoot dry weight, root dry weight (*n* = 3 plants per biological replicate), and leaf relative water content (*n* = 3–4 biological replicates). Additionally, the abundance of ^15^NH_4_, ^15^NO_3_, ^14^N (*n* = 3 biological replicates), and *δ*^13^C isotopes (*n* = 3 biological replicates and 2 technical replicates) were measured by mass spectrometry (UC Davis Stable Isotope Facility) (Supplementary Fig. 2).

To detect transcriptional responses after short exposure times to N-treatment (Fig. 3), we employed a hydroponics setup. Specifically, we grew rice seedlings (*Nipponbare*) hydroponically in 200 mL of W in Murashige and Skoog media^37^ holding an N-concentration of 1 mM KNO_3_ for 10 days. We then excised remaining endosperm and starved plants for N for an additional 3 days. On day 14, plants were transferred to a solution of 200 mL W with an N-concentration of 20 mM KNO_3_ and 20 mM NH_4_NO_3_^37^ 2 h after subjective dawn. Plants were treated for 0, 5, 10, 15, 20, 30, 45, 60, 90, and 120 min exposure times to N (*n* = 3 biological replicates per condition). Additionally, we treated plants over the same time periods with 20 mM KCl as a control treatment (*n* = 3 biological replicates per condition). One hundred percent of N ions within the treatment were labeled with ^15^N. After treatment, a portion of rice shoots and roots were sampled for RNA-seq and for mass spectrometry.

For our field experiments, 19 rice cultivars (listed in Supplementary Fig. 5) were grown under field conditions at the IRRI at Los Banos, Philippines (July–December 2016). Each cultivar was supplied with either a N-replete dose of 150 kg/ha dose of (NH_4_)_2_SO_4_ or with no N-treatment, 23 days after sowing (DAS). Under each N condition, fields were either W-replete or W-deplete, the latter obtained by draining the field of W and protecting the field from rain (intermittent watering of W-deplete fields was required to sustain growth). For W-deplete conditions, the minimum soil water potential was −34 kPa (non N-fertilized) and −52 kPa (N-fertilized) at 74 DAS (as measured by tensiometers at 30 cm depth). For each N and W condition, rice cultivars were grown in triplicate in randomized block design, where each triplicate contained 20 plants.

For each of the 19 rice cultivars, leaf transcriptomes at 49 DAS were sampled at the vegetative stage from 2 individual rice plants in biological triplicate per condition, 2 h after dawn. Leaf tissue was stored in RNA later solution (Thermo Fisher Scientific) immediately upon sampling. Rice plants were sampled for shoot dry weight 49 DAS per field treatment per genotype (2 plants per biological triplicate). From vegetative rice samples, additional traits were measured as follows: W-use efficiency was measured from leaf tissue using *δ*^13^C isotopic discrimination by mass spectrometry (performed by IRRI Analytical Services Lab, pool of 3 plants per biological replicate). Tiller number was counted by hand from each vegetative sample (6 plants per treatment per genotype). Chlorophyll concentration index was measured 55 DAS (CCM-200 Chlorophyll concentration meter, Apogee Instruments, 2 plants per biological triplicate). Stomatal conductance was measured on two leaves per plot, averaged over two separate days, 45–48 DAS (AP4 porometer, Delta T Devices, 2 plants per biological triplicate). End-point phenotyping was measured as follows: Straw biomass was measured as total straw dry weight (g) from a plot divided by the sampling area (m^2^). Panicle number was recorded by hand from 6 plants per genotype per treatment. Days to flowering was counted as the length of time, in days from sowing, until half the plants in each replicate plot had visible panicle emergence. Plant height was measured from the base of the plant to the tip of longest leaf. Grain yield was measured as the aggregate grain amount per cultivar in each triplicate using formula (1):

1 $${\mathrm{grain}}\,{\mathrm{yield = (grain}}\,{\mathrm{weight}} \times {\mathrm{((100}}-{\mathrm{moisture}}\,{\mathrm{content)}}/{\mathrm{86))/sampling}}\,{\mathrm{area}}$$

We replicated the experiment for 14 out of the original 19 cultivars at IRRI the following year (July–December 2017). In this season, for W-deplete conditions, the minimum soil water potential was −27 kPa (low N) and −59 kPa (high N) at 73 DAS. We observed grain yield, vegetative biomass, final (straw) biomass, chlorophyll concentration, stomatal conductance, days to flowering, plant height, tiller number, and panicle number using the same techniques as the previous year. We then correlated these second season outcomes with that of the first (Supplementary Fig. 7). Additionally, at 49 DAS we collected leaf tissue samples for RNA-seq analysis from two cultivars IR-64 and IR83388-B-B-108-3, for transcriptomic analysis. We selected these lines because of their differing responses to N and W in the previous year’s sampling of 19 rice cultivars.

### Linear modeling and analysis of genome-wide data

RNA from all leaf tissue samples, collected under both laboratory and field conditions, was purified using the RNeasy Mini Kit (Qiagen) with on-column DNase treatment. RNA quality was assessed using Agilent Tape station using High Sensitivity RNA ScreenTape. A unit of 500–1 µg of total RNA per sample was depleted of rRNA by Thermo Fisher Scientific mRNA Purification Kit. All 411 RNA-seq libraries were made using the NEBNext Ultra RNA Library Prep Kit, and sequenced using Next Seq Illumina platform with 1 × 75 bp single read-end chemistry or the Illumina HiSeq 2500 v4 with 1 × 50 bp single-end read chemistry. Two libraries were excluded from the 4-by-4 factorial N-by-W treatment matrix, and one library was excluded from second season field testing described based on quality controls. Raw reads were trimmed to remove adapter and low-quality bases (*q* < 10). RNA-seq reads were aligned to the *Nipponbare* reference genome^38^ using BBMap. Read counts were calculated using HT-seq^39^.

From libraries generated from the 4-by-4 factorial N-by-W experiment we removed from our analysis genes with total read counts <128 (summed across all conditions) or a median read count <0 (across all conditions). Multivariate gene modeling on read counts for each of the remaining genes was performed in R, using DESeq2^15^, starting with the full generalized linear model (2):

2 $${\mathrm{gene}}_a\,{\mathrm{expression}} = {\mathrm{\alpha + \beta }}_1N{\mathrm{ + }}\beta _2W{\mathrm{ + }}\beta _3N/W + \beta _4N \times W$$

After the full linear model was fit to the RNA-seq read counts of each gene (using design ~*N* + *W* + *N*/*W* + *N*×*W*), we performed model simplification as follows: (1) for each gene, a false discovery rate (FDR)-adjusted *p*-value was computed for each of the factors within the model. (2) If a gene was fit significantly by all four terms (FDR-adj. *p* < 0.005 for all four factors), then this gene was deemed fit by the full model and removed from remaining steps. (3) For all remaining genes, the factor with the least significance (highest FDR-adj *p*) was removed, and the model was refit with the remaining terms. (4) If a gene was fit significantly by all three terms (FDR-adj. *p* < 0.005 for all three factors), then this gene was deemed fit by a three-term model and removed from remaining model simplification steps. (5) Steps 3 and 4 were repeated, fitting two-term and one-term models. If a gene was not fit by any model form, then it was removed from further analysis.

Normalized expression levels of each modeled gene was correlated with log_2_ shoot biomass, N-content, and W-use efficiency (Pearson correlation, FDR-adj. *p* < 0.05) (Fig. 1d). The number of genes that correlated with shoot biomass were 7, 469, 218, and 1071 for classes *N*, *W*, *N*/*W*, and *N*×*W*, respectively. The number of genes that correlated with N-content in were 1244, 0, 809, and 1427 for classes *N*, *W*, *N*/*W*, and *N*×*W*, respectively. The number of genes that correlated with W-use efficiency were 0, 215, 0, and 2 for classes *N*, *W*, *N*/*W*, and *N*×*W*, respectively. The number of genes that correlated with N amount were 1323, 0, 1425, and 1250 for classes *N*, *W*, *N*/*W*, and *N*×*W*, respectively. The number of genes that correlated with W were 0, 1071, 879, and 893 for classes *N*, *W*, *N*/*W*, and *N*×*W*, respectively (Supplementary Fig. 3). Orthologs of rice genes were computed using OrthoMCL^40^. GO term enrichment analysis was performed by first converting rice genes to their *Arabidopsis* orthologs using OrthoMCL^40^. GO term analysis for each orthologous gene response class was performed in rice VirtualPlant^41^, using the genes expressed within the N-by-W factorial experiment as the background set. The full list of significant GO terms can be found in Supplementary Data 1. To understand whether gene expression responses to changes in *N*-moles and *N*-molarity was a binary response (Fig. 1e), we computed the ratio between normalized *N* and *N*/*W* coefficients for the genes fit by the *N*, *N*/*W*, and *N* + *N*/*W* gene models, and multiplied this value by 100 to obtain a percentage value. This value was deemed the weight that *N*-moles or *N*-molarity had on a gene’s expression. For example, a gene fit by the N model would have an N weight of 100% and an *N*/*W* weight of 0%. We computed the first principal component of *N*, *W*, *N*/*W*, and *N*×*W* gene classes, where reads were first logged to the base 2^22^. Principal components were calculated in R using the prcomp() function, and each resulting eigengene was then correlated to the dose of *N*, *W*, *N/W*, and *N*×*W* present, using Pearson correlation analysis (Supplementary Fig. 3).

Reads from libraries generated from the N-treatment time-series experiment in rice seedlings were normalized using DESeq2^15^. To detect genes that were differentially expressed in response to N-treatment over time, we fit log_2_-normalized gene expression patterns to both N-treated and KCl-treated samples using a cubic spline model (df = 5, 3 knots)^42^. Genes were deemed differentially expressed if the expression differences between N and KCl treatments over time were significantly different (FDR-adj. *p* < 0.05), and their combined read count across all time points was >100 reads. We then binned genes into the time points in which they were either activated or repressed. This was determined by assessing whether a gene’s average fold change expression at a given time point was >±1.25 when compared to the zero time point. We note that this approach allows a gene to be binned into multiple time points. We intersected these lists with genes modeled by N-dose response classes—*N*, *N*/*W*, and *N*×*W*—in our N-by-W factorial experiment in rice seedlings. The significance of each overlap was calculated using a Monte Carlo test with 10,000 permutations with an FDR correction. As background set for this analysis, we used the intersect of genes found expressed in our time-course analysis and within our 4 × 4 N-by-W factorial experiment.

For libraries generated from the field experiments, we removed from our analysis genes with total read counts below 128 (summed across all conditions) a median read count < 0 (across all conditions). A three-way analysis of deviance function with three categorical variables—W, N, and genotype—was called on read counts for each gene detected in field samples using DESeq2^15^ (design ~*N* + *W* + *N:W* + *genotype*). A gene was considered differentially expressed when either *N*, *W*, or *N*:*W* term within the model scored below FDR-corrected *p*-value of 0.05. We note the genotype factor was used to control for the effects between cultivars, but not used for sub-setting the data. A gene was binned as N-responsive or W-responsive when either respective term was significant, while the other term as well as the interaction term between N and W (*N:W*) was not (FDR-adj *p* > 0.05). If the interaction term was significant, a gene was binned as *N*×*W* when the N and W interaction term log_2_ fold change was positive, and binned as *N*/*W* when the log_2_ fold change was negative. Specifically, a positive log_2_ fold change indicated that differential gene expression occurred under high-W and high-N conditions, or low-W and low-N conditions. These genes were deemed *N*×*W* genes because this type of gene expression pattern agrees with *N*×*W* gene expression patterns found under laboratory conditions (where such genes were activated under high-N and high-W, or low-N and low-W treatments). The same logic applies to assigning *N*/*W* genes in the field. A negative log_2_ fold change value indicated that differential gene expression was driven by high-W and low-N conditions, or low-W and high-N conditions, a trend that *N*/*W* gene expression follows under lab conditions. We used Monte Carlo simulations to assess the significance of the overlap between classes of dose response genes identified in the laboratory and field: *N*, *W*, *N*/*W*, and *N*×*W*. As a background set for this analysis, we used the subset of genes found expressed under both lab and field conditions. Monte Carlo simulations were performed in VirtualPlant using the GeneSect function^41^. For heatmap visualization of lab-field validated gene sets, the expression value for each gene per cultivar in the field was first normalized to between 0 and 1, where 1 represented the maximum expression value (Fig. 4b).

We subsetted genes within each of the four classes—*N*, *W*, *N*/*W*, and *N*×*W*—to include only those genes that were consistently regulated across laboratory and field conditions (i.e. induced or repressed in both experiments). We then calculated the first principal components of each of the lab-field validated gene classes using the field gene expression data, logged to the base 2^22^. Each resulting eigengene was then correlated with log_2_ values of field phenotypes using Pearson correlation. Since direction of principal component eigenvectors are arbitrary with respect to sign, all correlation values are reported as positive correlations, regardless of the direction of the slope of correlation in plots. A *p*-value of the association between the eigengene and phenotype was calculated by comparison to a null distribution. This distribution was created by calculating an eigengene from a random gene set of the same sample size over 10,000 permutations. Only those eigengenes that passed a *p*-value cutoff of 0.05 were deemed significant.

To test whether *N*/*W* and *N*×*W* gene expression was indeed predictive of yield outcomes, we assessed whether eigengene expression of two cultivars (IR-64 and IR83388-B-B-108-3) sampled the following year was associated with yield results. To achieve this, we computed the first principal component from the expression of the 53 and 174 genes that made up the *N*/*W* and *N*×*W* eigengene classes, respectively. We then correlated resulting eigengenes genes with yield outcome using Pearson correlation (Fig. 4e, Supplementary Fig. 6). A *p*-value of the association between the eigengene and phenotype was calculated by comparison to a null distribution of 10,000 random eigengenes created from expressed genes in the field. This eigengene analysis indicated that resulting associations were significant (permutation test, *p* < 0.05).

### Reporting summary

Further information on experimental design is available in the Nature Research Reporting Summary linked to this article.

## Supplementary information

Supplementary Information

Reporting Summary

Description of Additional Supplementary Files

Supplementary Data 1

Supplementary Data 2

Supplementary Data 3

Supplementary Data 4

Supplementary Data 5

Source Data

## Data Availability

Data supporting the findings of this work are available within the paper and its Supplementary Information files. A reporting summary for this article is available as a Supplementary Information file. R codes that support the findings of this study and the data sets generated and analyzed during the current study are available from the corresponding author upon reasonable request. Sequencing data can be found at the National Center for Biotechnology Information Sequence Read Archive, with Accession number PRJNA510920. The source data for Figs. 1d, 2, 3, and 4b–e, and Supplementary Figures 1A and 2–4 are provided as a Source Data file.
