# Supplementary material for: Water impacts nutrient dose responses genome-wide to affect crop production
Source: Nat Commun. 2019 Mar 26;10:1374. doi: 10.1038/s41467-019-09287-7 (PMC6435674; doi:10.1038/s41467-019-09287-7)
Supplement: Supplementary file 1 — Supplementary Information [file 41467_2019_9287_MOESM1_ESM.pdf]

Water impacts nutrient dose responses genome-wide to affect crop production

Swift *et al.*

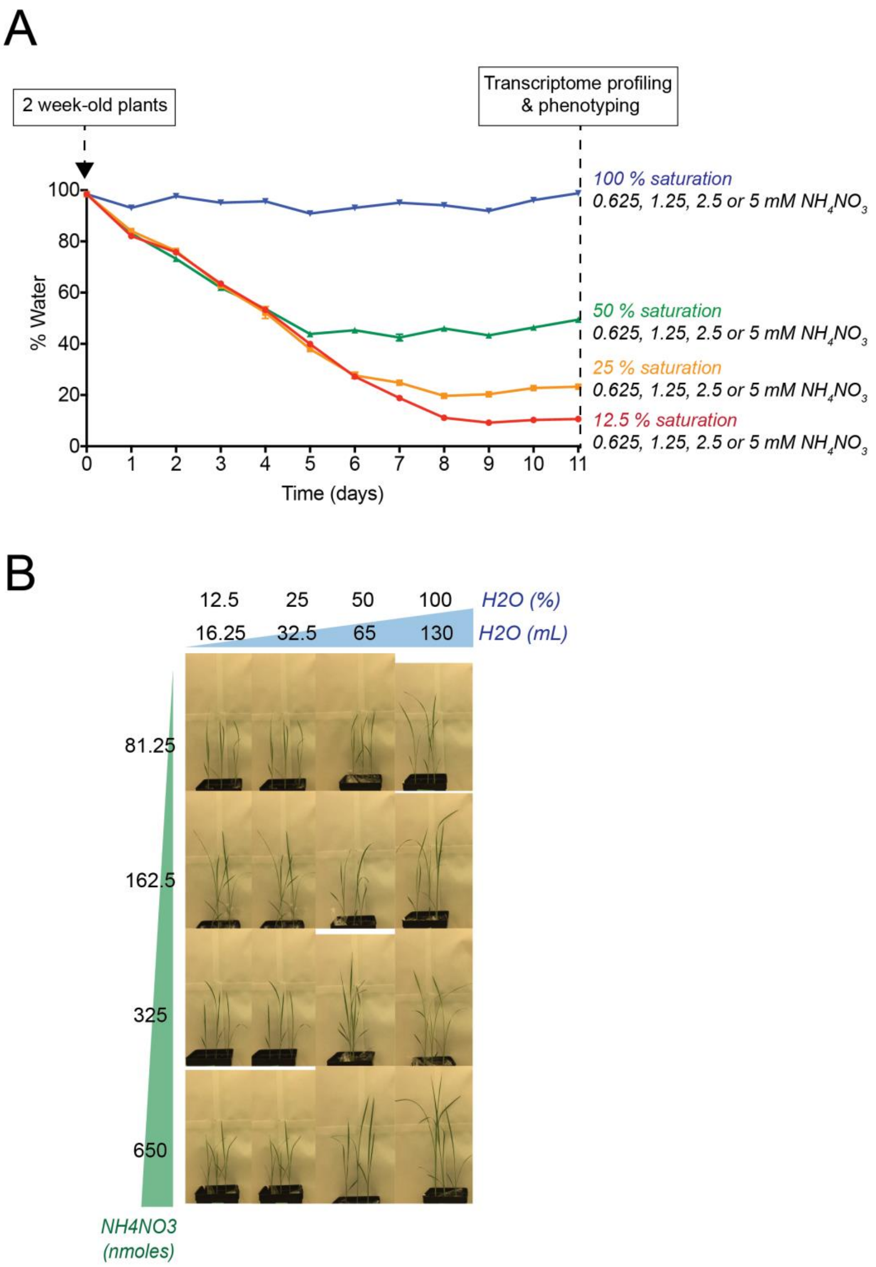

Supplementary Figure 1. Execution of the 4-by-4 factorial N-by-W matrix. A: The 4-by-4 factorial N-by-W treatment matrix for rice seedlings varied both N and W amounts. To create these conditions, all treatment pots began at 100% W saturation with different N-concentrations. Evaporation was allowed to occur over time until each pot reached the desired W level. W was then maintained at the desired saturation level through daily additions of W. Each pot condition was replicated in triplicate, resulting in 12 pots per W condition (error bars indicate SEM). **B:** Images of plants grown within the N-by-W factorial treatment matrix. Images were taken on the 11th day of treatment. Supplementary Figure 1A source data is provided in Source Data file.

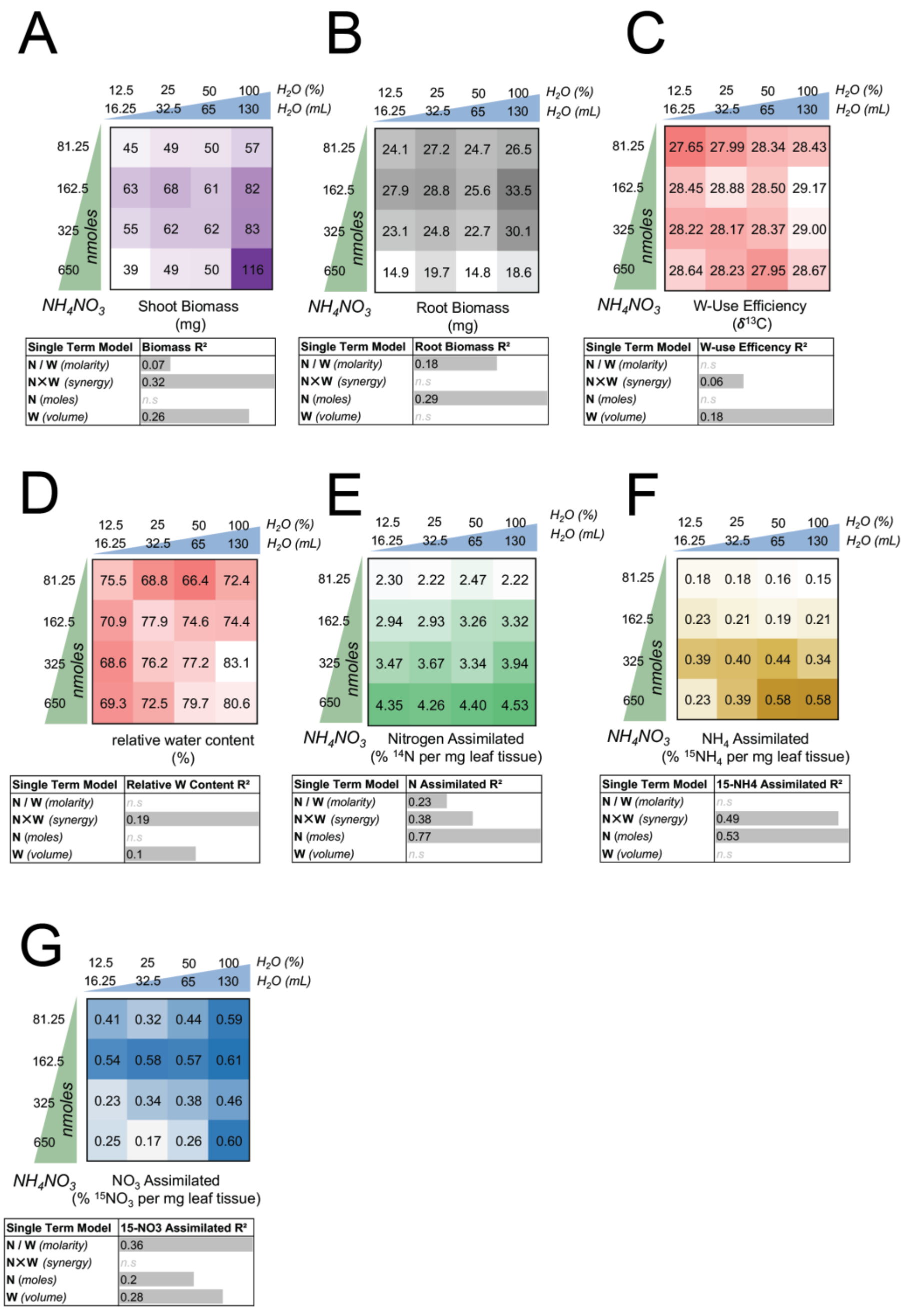

Supplementary Figure 2. Measuring the effects of combinations of N-moles and W-volume on rice seedling phenotypes. A range of phenotypes were measured from rice seedlings grown under the experimental N-by-W design matrix treatment. We tested the ability for one of four linear models, each holding a single term, to explain phenotype. When a model significantly explained phenotype (linear model, $p < 0.05$), the resulting R^2 is provided. We performed this analysis for **A:** shoot dry weight **B:** root dry weight **C:** water use efficiency **D:** leaf relative water content **E:** percent of total ^{14}N assimilated in leaf tissue **F:** percent of $^{15}NH_4$ assimilated in leaf tissue **G:** percent of $^{15}NO_3$ assimilated in leaf tissue. Supplementary Figure 2 source data is provided in Source Data file.

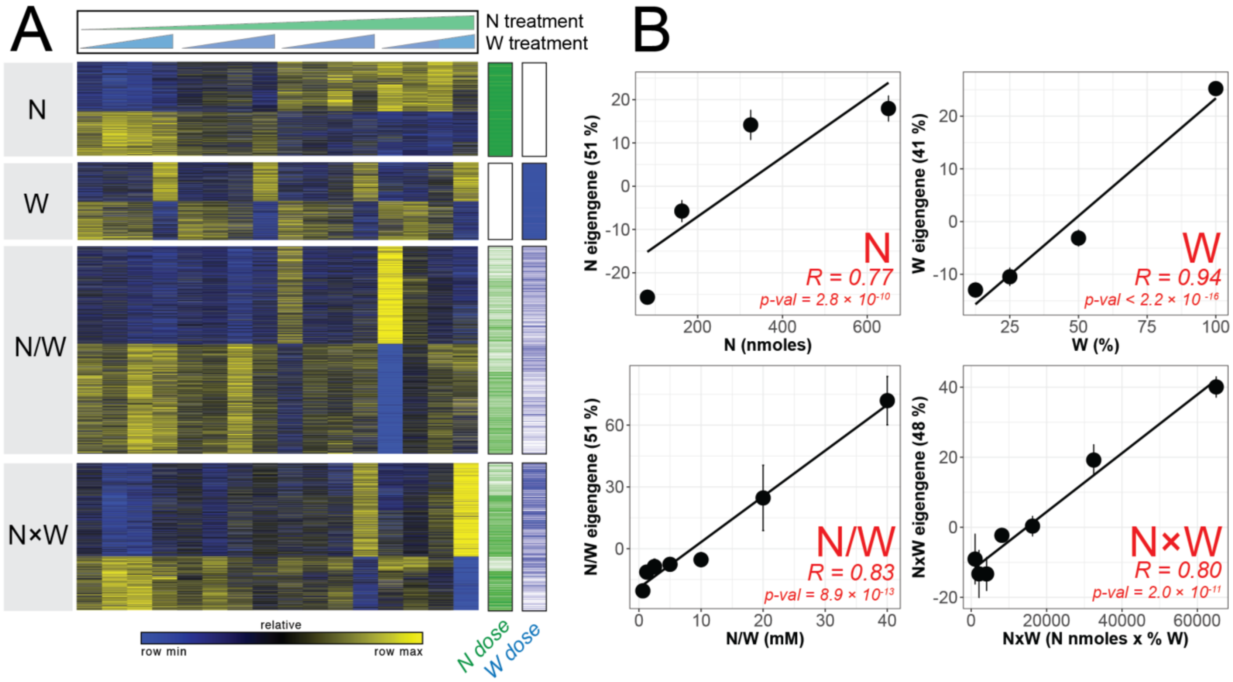

Supplementary Figure 3. Correlating *N*, *W*, *N/W* and *N*×*W* gene classes with N and W amounts supplied to plants. A: Expression heatmap of genes fitted by a single model term, and the proportion of genes within each class that significantly correlated with the amount of N or W provided (Pearson correlation, FDR-adj. $p < 0.05$). **B:** The first principal component, or 'eigengene', of each gene class correlated significantly with the dose of N, W, N/W or N×W present. Pearson R values, significance of association, and percent variance each principal component explained are provided (error bars indicate SEM). Supplementary Figure 3 source data is provided in Source Data file.

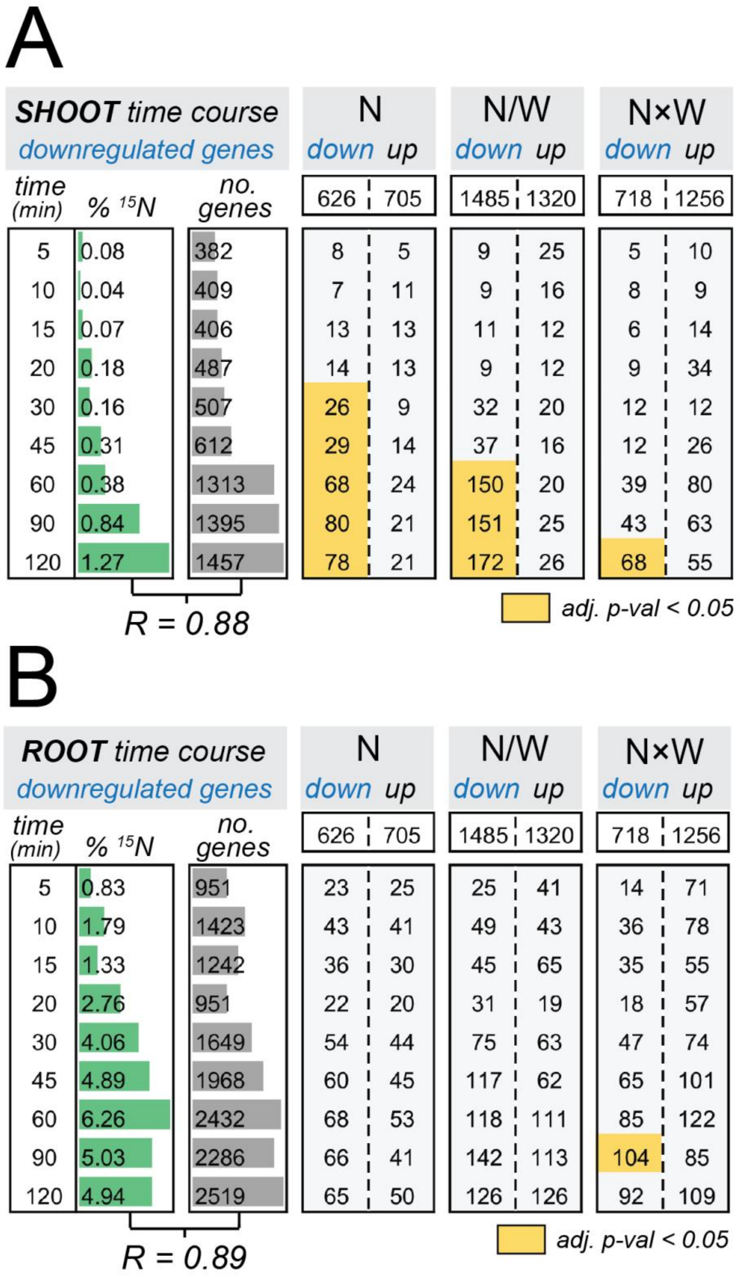

Supplementary Figure 4. *N*-moles, *N*/*W* (N-molarity) and *N*×*W* gene classes respond rapidly to a change in N-dose in root and shoot tissue. A: Rice seedlings were treated with N for 120 min (see Methods). During this period, we monitored shoot gene expression responses and shoot N-uptake via ^{15}N labelling. N-uptake significantly associated with the total number of differentially repressed genes (Pearson R = 0.88, $p = 1.7 \times 10^{-3}$). *N*, *N*/W and *N*×*W* gene classes that were downregulated in response to N-dose within our factorial matrix experiment intersected significantly with genes downregulated in response to a change in N-dose (Monte Carlo, FDR- adj. $p < 0.05$) **B:** The same analysis performed in root tissue (Pearson R = 0.89, $p = 1.1 \times 10^{-3}$). Supplementary Figure 4 source data is provided in Source Data file.

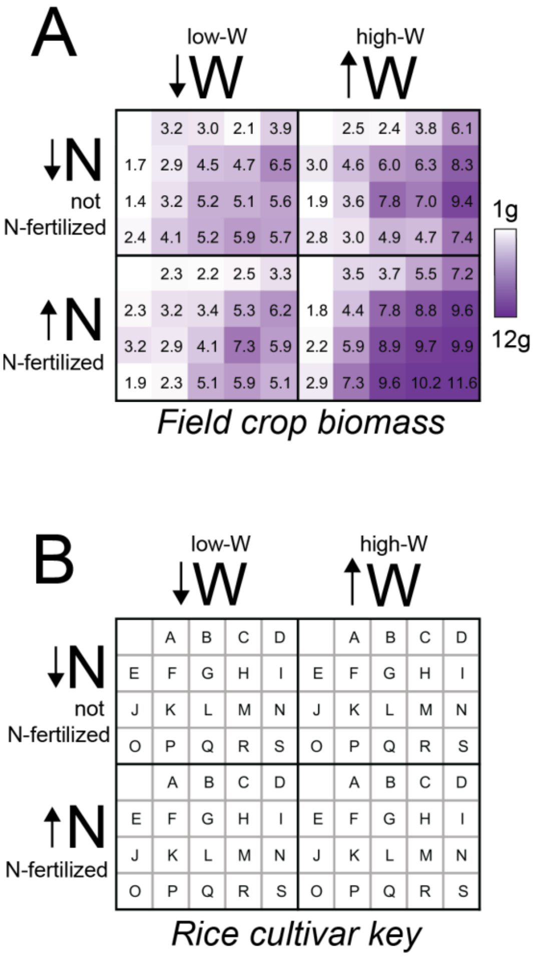

C

Key	Cultivar	Subgroup
A	Haginomae Mochi	Japonica
B	Nipponbare	Japonica
C	PR106	Indica
D	BG90-2	Indica
E	Tainung 67	Japonica
F	Beonjo	Japonica
G	IR20	Indica
H	IR83388-B-B-108-3	Indica
I	IR83383-B-B-129-4	Indica
J	Palawan	Indica
K	BG34-8	Indica
L	PSBRC 82	Indica
M	IR74371-54-1-1	Indica-Japonica
N	IR87707-445-B-B-B	Indica
O	Yabani Montakhab	Japonica
P	BG380-2	Indica
Q	IR64	Indica
R	IR74371-70-1-1	Indica-Japonica
S	IR83380-B-B-124-1	Indica

Supplementary Figure 5. List of 19 rice cultivars grown under field conditions. A: Field crop biomass of 19 rice cultivars was recorded 49 days after sowing (reproduced from **Fig. 4A**). The position of each cultivar is provided in **B**, and their full name and subspecies is provided in **C**. Cultivars were chosen based on reports of being N-use efficient or W-use efficient either in literature or from prior field observations.

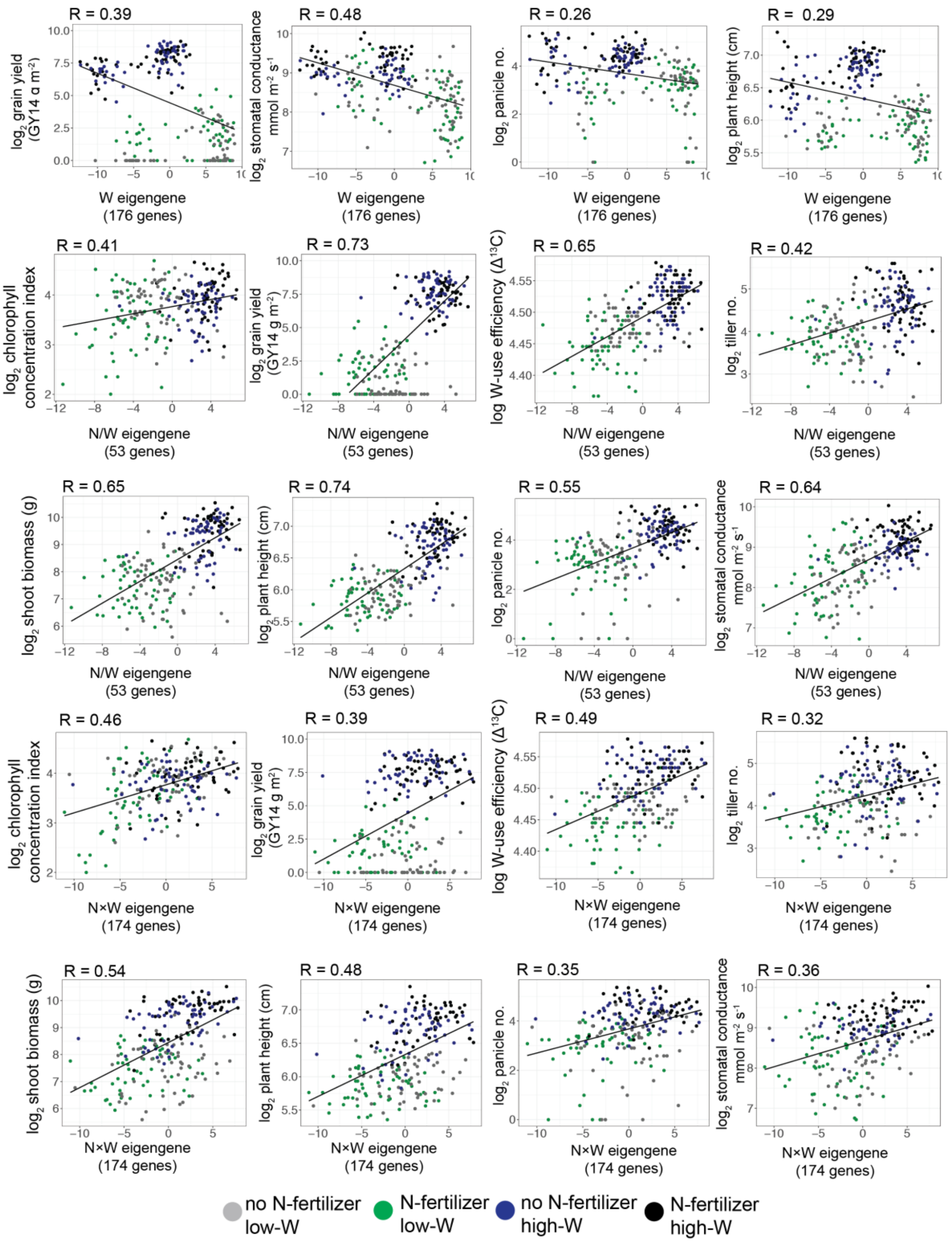

Supplementary Figure 6. Correlating lab-field validated eigengenes with field phenotypes. For each of our lab-field validated gene sets responding to N-moles (56 genes) W-volume (176 genes), N-molarity (*N*/*W*) (53 genes), or a synergistic response to *N*×*W* (174 genes), we reduced the expression trends of all gene members into a single profile or 'eigengene' (grey: -N, -W, green: +N, -W, blue: -N +W, black: +N,+W conditions). We then correlated each eigengene with field phenotypes. The significance of association was calculated by comparison to a null distribution of 10,000 random eigengenes. Significant associations (permutation test, $p < 0.05$) are shown here, with significant R values (Pearson correlation, $p < 0.05$) displayed.

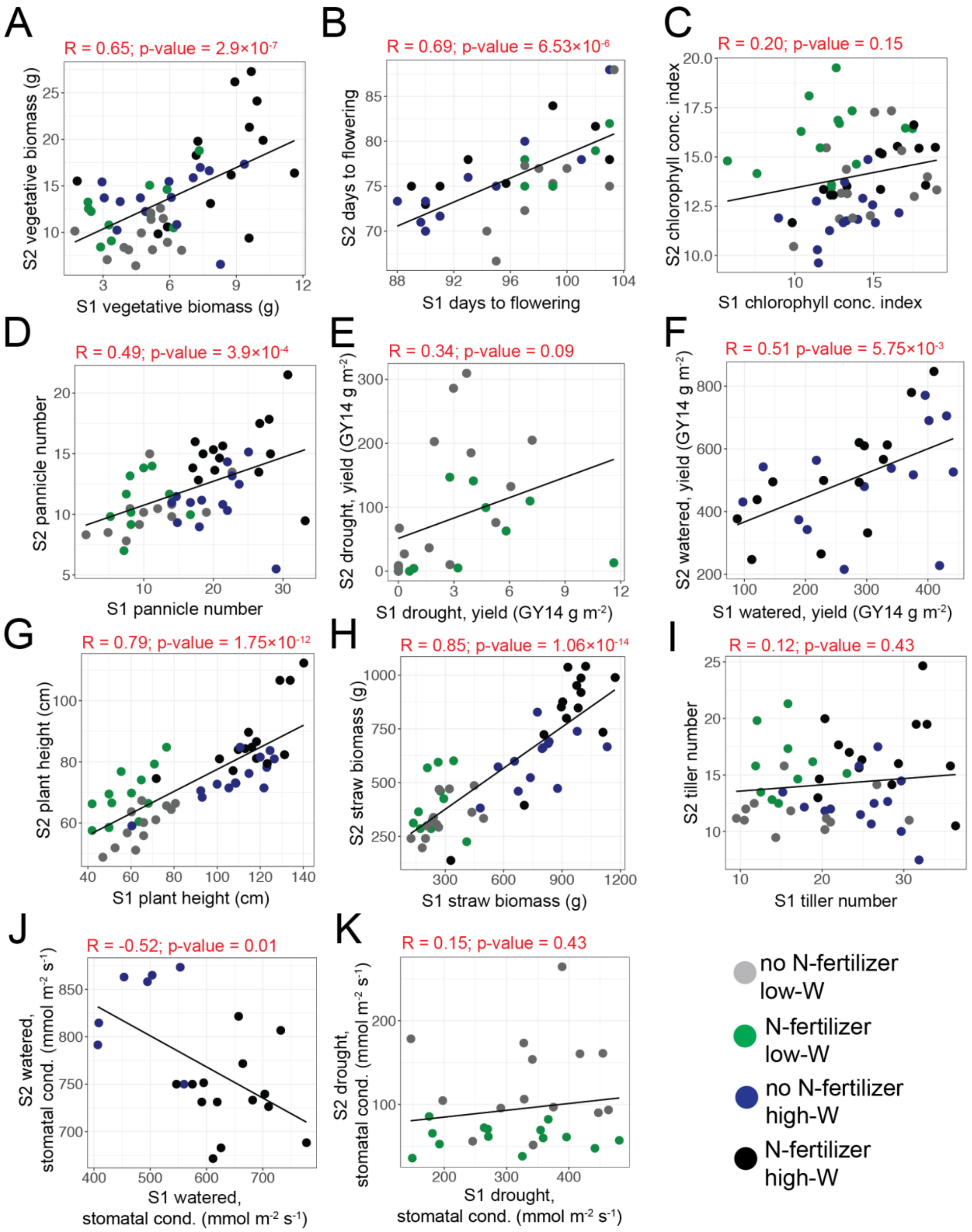

Supplementary Figure 7. Testing reproducibility of field phenotypes. For 14 of the 19 cultivars tested, we duplicated our field experiments at the International Rice Research Institute in the Philippines (July – December 2017). We found that phenotypes between the 2016 ('S1') and 2017 ('S2') seasons were largely reproducible, as demonstrated through Pearson correlation analysis. Grain yield (**E** and **F**) and stomatal conductance (**J** and **K**) observations were separated into well-watered or drought treated before correlation analysis (grey: -N, -W, green: +N, -W, blue: -N +W, black: +N,+W conditions).

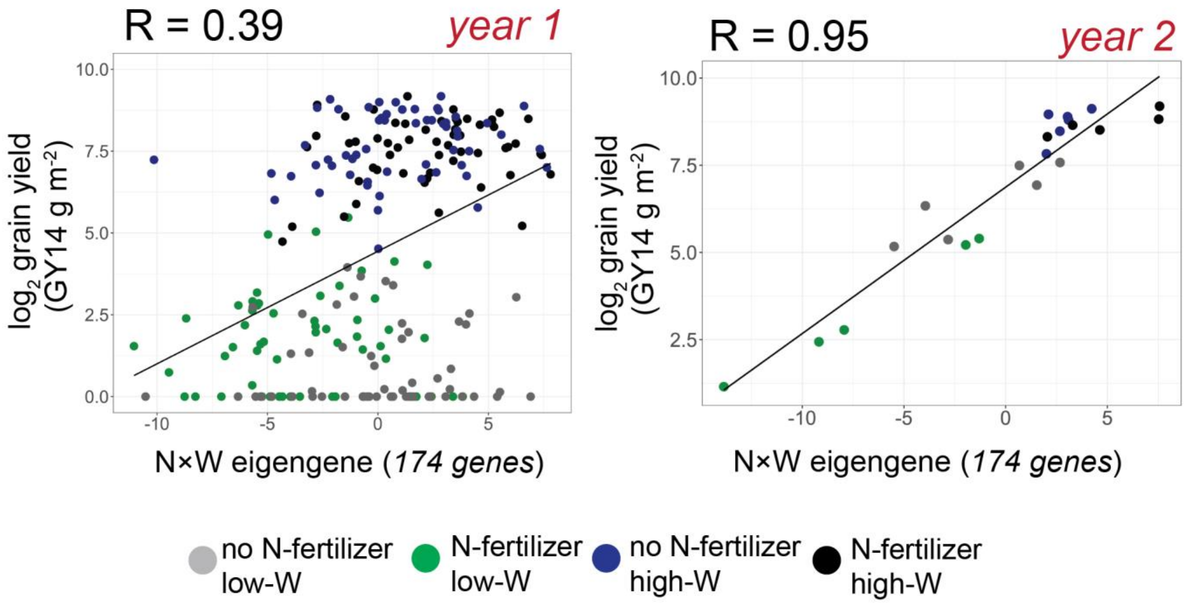

Supplementary Figure 8. *N*×*W* eigengene expression is predictive of crop outcome measures within an independent, replicated field test. A: Changes in *N*×*W* eigengene expression across 228 field samples is significantly associated with grain yield (permutation test, $p < 0.05$, grey: -N, -W, green: +N, -W, blue: -N +W, black: +N,+W conditions). **B**: *N*×*W* eigengene expression is predictive of grain yield within an independent field test set observed the following year (permutation test, $p < 0.05$).
