# Supplementary material for: Water impacts nutrient dose responses genome-wide to affect crop production
Source: Nat Commun. 2019 Mar 26;10:1374. doi: 10.1038/s41467-019-09287-7 (PMC6435674; doi:10.1038/s41467-019-09287-7)
Supplement: Supplementary file 3 — Description of Additional Supplementary Files [file 41467_2019_9287_MOESM3_ESM.docx]

**Description of Additional Supplementary Files**

File Name: Supplementary Data 1

Description: Genes significantly regulated in response to changes in N and/or W dose in rice seedlings under lab conditions

File Name: Supplementary Data 2

Description: Significant GO Terms associated with N, W, N∕W and N×W gene classes in rice seedlings

File Name: Supplementary Data 3

Description: Genes significantly regulated by a change in N-dose over a 120 min time span

File Name: Supplementary Data 4

Description: Genes significantly regulated in response to N and/or W-dose treatments in rice plants grown under field conditions

File Name: Supplementary Data 5

Description: List of rice genes responsive to changes in N, W, N/W or NxW doses under both laboratory and field conditions
